# Structural and Psychometric Properties of Neck Pain Questionnaires Through Patient-Reported Outcome Measures: A Systematic Review

**DOI:** 10.3390/medicina61071254

**Published:** 2025-07-10

**Authors:** Manuel Gonzalez-Sanchez, Álvaro Jesús Reina-Ruiz, Guadalupe Molina-Torres, Sandra Kamila Trzcińska, Elio Carrasco-Vega, Alena Lochmannová, Alejandro Galán-Mercant

**Affiliations:** 1 Department of Physiotherapy, Faculty of Health Sciences, University of Málaga, Avda. Cervantes, 2, 29071 Malaga, Spain; mgsa23@uma.es (M.G.-S.); eliotafad@hotmail.com (E.C.-V.); 2 IBIMA—Instituto de Investigación Biomédica de Málaga, 29010 Malaga, Spain; 3 Department of Nursing, Physical Therapy and Medicine, Faculty of Health Sciences, University of Almería, Carretera de Sacramento s/n, 04120 Almería, Spain; guada.lupe@ual.es; 4 Department of Clinical Physiotherapy, Józef Piłsudski University of Physical Education in Warsaw, 00-968 Warsaw, Poland; sandra-trzcinska@wp.pl; 5 Department of Paramedic Science, Medical Diagnostics and Public Health, Faculty of Health Care Studies, University of West Bohemia, 301-00 Pilsen, Czech Republic; lochmann@fzs.zcu.cz; 6 Institute of Biomedicine of Cádiz (INIBICA), 11009 Cadiz, Spain; alejandro.galan@gm.uca.es; 7 MOVE-IT Research Group, Department of Nursing and Physiotherapy, Faculty of Health Sciences, University of Cádiz, 11009 Cadiz, Spain

**Keywords:** psychometric properties, structural characteristics, questionnaire, neck pain, patient-reported outcome measures

## Abstract

*Background and Objectives*: Questionnaires are patient-reported outcome measures that require a validation process to assess their reliability and replicability. Over time, questionnaires have not only focused on a single health condition, such as neck pain, but also expanded their assessment spectrum to other areas in order to gather additional and relevant information from the patient. The main objective of this study was to conduct a systematic review of the different structural and psychometric characteristics of neck pain questionnaires. *Materials and Methods*: A systematic review was conducted following the PRISMA recommendations. The search strategy was implemented across various databases (PubMed, Cochrane, EMBASE, CINHAL, Trip Medical Database, Scopus) using terms such as neck pain, cervicalgia, cervical pain, questionnaire, survey, index, validity, validation, and reliability. COSMIN criteria were used to identify valid questionnaires for this systematic review based on their psychometric properties. *Results*: A total of 15 articles were identified in this systematic review, of which 8 assessed the level of disability, while the rest evaluated dizziness in neck pain, anxiety and/or depression, beliefs about fear and avoidance, and perception of scarring and symptoms after neck surgery. The main findings show that neck pain questionnaires exhibit very good values for reliability and internal consistency, along with a high variability for construct validity. *Conclusions*: This study highlights the good values exhibited by neck pain questionnaires despite their heterogeneity in structural characteristics, demonstrating good values in psychometric properties. Nevertheless, the latter should be further investigated to gather more information.

## 1. Introduction

Neck pain is one of the most prevalent musculoskeletal pathologies in the healthcare system [1]. A large percentage of cases are considered of unknown etiology, resolving in many instances within the first few weeks [2]. However, in the remaining cases, individuals often experience pain for more than 3 months, leading to chronicity [3]. This health condition usually carries a significant impact both individually and globally, as well as an economic repercussion [4], with a significant increase in the percentage of affected people from 60.2 to 115.9% in disability-adjusted life years (DALYs) between 1999 and 2019 [5].

A questionnaire is a patient-reported outcome measure (PROM) that provides direct information about health variables such as symptoms, quality of life, and functionality [4]. In addition, both the original and translated versions in other languages must undergo a validation process of their psychometric properties, mainly validity and reliability; subsequently, it is necessary to test their internal consistency, test-retest reliability, and inter-rater reliability, to ensure that the collected results are reliable and can be replicated over time [5]. All data filled from a questionnaire can lead to better decision-making, advancement in knowledge, and improvement in clinical practice by the professional applying it with their patients [6].

Likewise, questionnaires in the physiotherapy setting aim to follow the principles of the International Classification of Functioning, Disability and Health (ICF), integrating a psychosocial perspective into the current biomedical model in some way as a reference guide for the standardization of measurement tools [7]. Lately, questionnaires are not only focusing on the effectiveness of certain treatments for a health condition in a specific population, but they are also extending to the clinical setting with the aim of improving communication between the patient and the professional, as well as patient satisfaction [8]. Moreover, one of the tools used for the assessment of PROMs in systematic reviews is the COnsensus-based Standards for selecting health Measurement Instruments (COSMIN) checklist, which assesses the methodological quality of studies focusing on the measurement properties of the evaluation questionnaires and their own validation [9].

Despite the existence of systematic reviews focused on the analysis of the psychometric characteristics of a specific issue to assess disability in patients with neck pain, such as the Neck Disability Index (NDI) [10,11], there is a clear lack of studies that systematically analyze the structural characteristics and psychometric properties of questionnaires aimed at assessing and monitoring patients with neck pain in all areas, not only for pain outcome. Studies on this topic would allow both the clinical and research scopes to have the best assessment tools based on their results. The objective of this systematic review was to assess the different structural characteristics and psychometric properties of the questionnaires that are currently used to measure neck pain. As a secondary objective, this study aimed to provide guidelines that can assist in selecting the questionnaire best suited to the needs and clinical or research reality of anyone requiring the use of one of the selected tools.

## 2. Materials and Methods

### 2.1. Protocol

This systematic review followed the general guidelines and recommendations of the Preferred Reporting Items for Systematic Reviews and Meta-analyses (PRISMA) [12]. Additionally, it was registered in PROSPERO with the following registration number: CRD42024509659.

### 2.2. Sources and Search

For this systematic review, articles were searched in the following databases: PubMed, Cochrane, EMBASE, CINHAL, Trip Medical Database, and Scopus.

The article search was focused on questionnaires related to neck pain. The following keywords, based on MeSH headings and Entry Terms, were used along with Boolean operators “OR” and “AND” without applying any search filters, except for Embase and Scopus databases (Supplementary Table S1): neck pain, cervicalgia, cervical pain, whiplash injuries, questionnaire, survey, index, checklist, validity, validation, and reliability. One of the search strategies employed for all databases was: (neck pain OR neckache or neck injur* OR cervicalgia OR cervical pain OR whiplash injuries) AND (questionnaire OR survey OR index OR scale OR tool OR inventory OR instrument OR score OR checklist OR PROM OR Patient Reported Outcome Measure) AND (validity OR validation OR reliability OR psychometric properties OR clinimetric*).

### 2.3. Eligibility Criteria

The inclusion criteria used for this systematic review were: validation studies of questionnaires focused on neck pain and characteristics of the psychometric variables of neck pain questionnaires in their original version. Studies published in languages other than English, Italian, and Spanish were excluded, as well as validation studies of questionnaires that have not been originally developed in English, Italian, or Spanish. All articles published by 31 January 2025 were considered eligible.

### 2.4. Study Selection

The article selection process was carried out by two independent researchers. After the bibliographic search, articles were filtered based on title and abstract. Once this process was completed, the documents were read in-depth to determine their eligibility for the study based on inclusion and exclusion criteria. In case of discrepancies among authors, a third author decided whether the article would be included.

### 2.5. Synthesis of Results and Data Extraction

From each selected article, both structural characteristics (full name, acronym, number of items, sub-categories, how long it takes to complete, resulting scale, number of versions and cost) and psychometric properties were extracted: reliability (test-retest reliability, internal consistency, and Standard Error of Measurement (SEM)), which is the degree to which the measurement is free from error; validity (content validity (including face validity), structural validity, hypotheses testing for construct validity, cross-cultural validity, and criterion validity), which is the proportion of variance in measurement due to real differences among patients; and responsiveness, which is the PROM’s ability to detect long-term changes in the evaluated variable [9].

### 2.6. Quality Assessment of the Questionnaires Included

To assess the quality of each of the questionnaires included in this systematic review, the COnsensus-based Standards for selecting the health Measurement Instruments (COSMIN) scale was used, which generates taxonomic consensus according to the different psychometric properties used in assessment instruments [13]. This scale typically evaluates four domains: validity, responsiveness, reliability, and interpretability, where higher values on a scale of 0 to 10 indicate better-evaluated tools (Supplementary Table S2).

The quality of the questionnaires included in this study was evaluated by two independent reviewers, and, in case of disagreement, consensus was reached among the other authors involved in this systematic review. Correlation between reviewers was measured using the Kappa index.

#### 2.6.1. Test-Retest Reliability

The confidence interval (CI) is a measure of reliability where its variance depends on the objects of measurement divided by the total variance [14]. It is considered adequate or inadequate when its score is >0.7 or <0.7, respectively, and the use of Pearson’s and Spearman’s correlations is not adequate due to systematic errors [14].

##### Internal Consistency

The internal consistency of the measures was calculated using Cronbach’s α. To classify both measures, the following scale was used: excellent (≥0.80); good (0.60–0.80); moderate (0.40–0.60), and poor (≤0.40) [15].

#### 2.6.2. Construction Validity

Construction validity refers to the degree of agreement of a PROM score with the stated hypothesis, such as the relationship with other instruments, to verify whether it actually measures what it proposes to measure [16]. If the result is positive, it is in agreement with the hypothesis; if it is negative, it is in disagreement with the hypothesis; and if it is neither positive nor negative, the hypothesis should be reconsidered [16].

#### 2.6.3. Factor Analysis

The factor analysis is considered the previous step to evaluate the internal consistency of a PROM, since, through the analysis of each of the items that make it up and the relationship between each of them, it defines the reflection of the dimensionality of the construct to be evaluated [16]. The result is considered negative or doubtful when no data are presented or remain to be defined [16].

#### 2.6.4. Sensitivity

Sensitivity refers to the number of individuals who obtain a positive result in a test, i.e., detecting affected people, and is generally considered to be of acceptable validity when its score is equal to or higher than 0.8 [17].

#### 2.6.5. Standard Error of Measurement

The Standard Error of Measurement is considered a reliability tool that measures detectable changes, and its calculation depends on the standard deviation and reliability of the measurement instrument, whose formula is the following:

SEM = SD √(1 − R),

where the value of 1 SEM can be equivalent to 0.5 standard deviations when the reliability of the instrument is 0.75 [18].

#### 2.6.6. Minimal Detectable Change

The minimum detectable change (MDC), also referred to as the smallest detectable change, is based on test-retest reliability and shows those changes that fall outside the measurement error through the following formula:

MDC = 1.96 × √2 × SEM,

representing that 2 measurements are involved under a 95% confidence interval of no change [18].

#### 2.6.7. Minimal Clinically Important Change

Minimal clinically important change (MCIC) is a completely different measure from MDC that focuses on the minimal changes reported by patients, clinicians, or other important stakeholders [18]. The cutoff point will depend on the definition used by the author of the study on the anchor, where, for some, it will be ‘a good deal better’ and, for others, it will be ‘somewhat better’ [18].

## 3. Results

**Figure 1:**
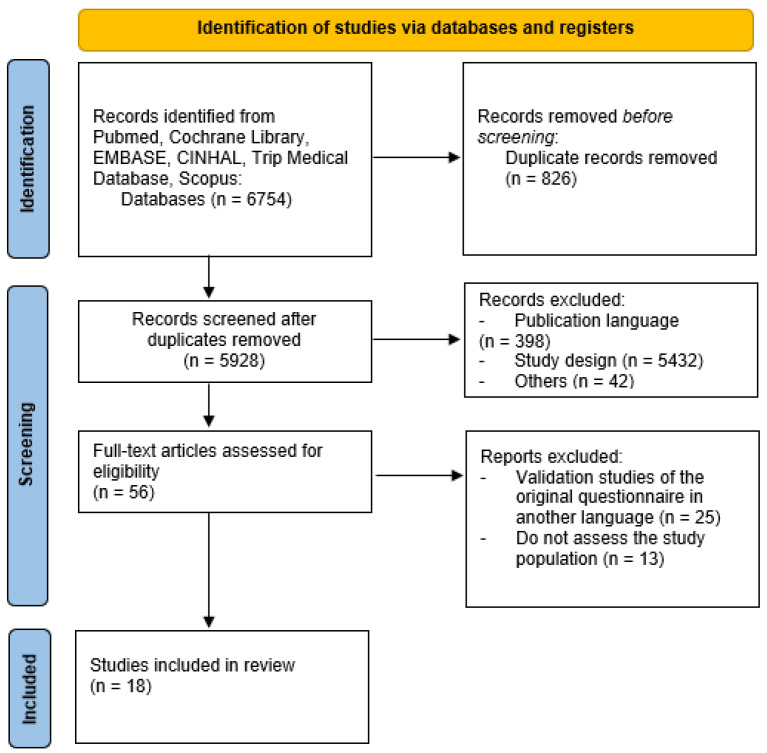
CONSORT flowchart diagram.

Initially, a search was carried out in the different databases, where a total of 6754 articles were identified, of which 826 were excluded due to duplication. Subsequently, after reading the title and abstract, a total of 5928 articles were filtered based on the inclusion and exclusion criteria established regarding publication language, study design, and article accessibility, leaving 56 articles available for evaluation, of which 18 were finally included in the study, representing 3.75% of the total. Correlation between reviewers obtained a value of 0.988 on the Kappa index. Figure 1 shows the flowchart with the screening and selection process of the different questionnaires.

### 3.1. Structural Variables

Table 1 shows the structural characteristics of all the questionnaires identified for neck-pain evaluation (*n* = 18) with a general sample of 4326 subjects, where the smallest sample size was 44 patients [19] and the largest was 944 patients [20]. The minimum and maximum ages were 18 [21] and 55 years [22], respectively. In relation to the number of items, great heterogeneity was found, ranging from 5 [21] to 44 items [23]. Ten of these questionnaires presented subscales [20,21,23,24,25,26,27,28,29,30]. The only two questionnaires that reported on the time to complete were the Functional Rating Index [25], with an average completion time of 78 s, and the Fear-Avoidance Beliefs Questionnaire, with a mean of 5 min. According to the assessment of every questionnaire, lower values indicate better results, with the exception of the Dizziness Handicap Inventory [24], the Patient Scar Assessment Questionnaire [29], and the Profile Fitness Mapping neck Questionnaire (ProFitMap-neck) [23], where better status is associated with a higher result. All questionnaires are free to access, and the Neck Disability Index is the questionnaire with the largest number of versions [27]. Some of the questionnaires are related to neck pain, although their main aim is focused on assessing different items, such as the Fear-Avoidance Beliefs Questionnaire for pain-related anxiety [20] and the Hospital Anxiety and Depression Scale for detecting states of depression and anxiety [31].

### 3.2. Psychometric Variables

On the other hand, Table 2 shows the psychometric characteristics of the identified neck-pain assessment questionnaires. Except for four questionnaires, i.e., Hospital Anxiety and Depression Scale (HADS) [24], Patient Scar Assessment Questionnaire (PSAQ) [29], Neck Bournemouth Questionnaire (NBQ) [32], and Cervical Spine Outcomes Questionnaire [30], which present lower scores in test-retest reliability for at least one subscale, the vast majority present excellent ICC values (above 0.76). Likewise, the internal consistency of each questionnaire is excellent, with the exception of Neck Disability Index (NDI) [27], PSAQ [29], Northwick Park Neck Pain Questionnaire (NPQ) [19], and Neck OutcOme Score (NOOS) [28], whose Cronbach’s α values are between 0.60 and 0.80. The construct validity showed great variety, ranging from −0.65 [30] to 0.91 [29], using reference tools, such as NDI, Copenhagen Neck Functional Disability Scale (CNFDS), 11-point numerical rating scale, Owestry Disability Index, NPQ, The Global Perceived Effect, Numeric Pain Rating Scale, Tampa Scale for Kinesiophobia-11 items version, Pain Catastrophizing Scale, and Short Form-36 Health Survey (SF-36), for some questionnaires. A factor analysis was carried out for three questionnaires, ranging from 7.4% [20] to 61.6% [33], where two questionnaires had four factors assessed [20,34]. The percentages explain the variances of the factors that were selected by the authors, i.e., the Exploratory Factor Analysis reflects the dimensionality of the assessed construct.

However, a negative aspect that needs to be highlighted is that psychometric characteristics have either not been calculated at all or have only been calculated by a very limited number of PROMs. In this regard, sensitivity was not evaluated in any of the questionnaires, and seven questionnaires had their SEM assessed, with values between −1.25 [28] and 9.54 points [35]. Three questionnaires show values of minimum detectable change between 0.12 points [23] and 21.41 points [36]. Lastly, two questionnaires focus on minimal clinically important changes, with 2.67 points [35] and 18 points [24], respectively. The SEM values, minimum detectable change, and minimal clinically important differences are based on the 95% CI of the total score.

### 3.3. COSMIN Checklist Analysis

Table 3 presents the evaluation of all dimensions proposed by the COnsensus-based Standards for selecting Health Measurement Instruments (COSMIN). The selected Patient-Reported Outcome Measures (PROMs) achieved scores ranging from 2 [26,36] to 7 [20]. All PROMs had their reliability calculated, and the vast majority also present criterion validity. However, none of the PROMs report their content validity.

**Table 1:** Descriptive characteristics of the sample and structural characteristics of the questionnaires in different versions for neck-pain assessment.

Questionnaire	Acronym	Population		Nº Items	Sub-Category	Time to Complete	Measurement (Best-Worst)	Versions	Cost
		N	Age						
5-item version of the Neck Disability Index [21]	NDI-5	316	18–70 years	5	5 Personal care; Concentration Work; Diving Recreation	-	0–100	1	-
Cervical Spine Outcomes Questionnaire [30]	CSOQ	216	49.39 ± 10.15 years	20	6 Neck pain severity Shoulder-arm pain severity Functional disability Phsycological distress Physical symptoms Health Care Utilization	-	0–100	1	-
Copenhagen Neck Functional Disability Scale [37]	CNFDS	162	38–56	15	0	-	0–30	9	-
Dizziness Catastrophizing Scale [33]	DCS	457	53.4 ± 15.4 years	13	0	-	0–52	2	-
Dizziness Handicap Inventory [24]	DHI	63	49.4 ± 18.5 years	25	3 Functional; Physical Emotional	-	100–10	14	-
Fear-Avoidance Beliefs Questionnaire [20]	FABQ	N_1_ = 247 N_2_ = 220 N_3_ = 139 N_4_ = 338	44.64 ± 9.83 years 42.81 ± 9.99 years 43.06 ± 9.99 years 42.01 ± 9.51 years	11	2 FABQPA: Fear-Avoidance Beliefs Questionnaire Physical Activity; FABQW: Fear-Avoidance Beliefs Questionnaire Work	5 ± 3 min	0–70	18	-
Functional Rating Index [25]	FRI	N_1_ = 150 N_2_ = 25	41 ± 15.8 years 46 ± 19.2 years	10	2 Function Pain	78 s	0–100	6	-
Hospital Anxiety and Depression Scale [26]	HADS	100	-	14	2 Anxiety Depression	-	0–21/each subscale	21	-
Neck Disability Index [27]	NDI	52	-	10	10 Pain intensity Personal care Lifting Reading Headache Concentration Work Diving Sleeping Recreation	-	0–100	31	-
Neck OutcOme Score [28]	NOOS	196	47.8 ± 13.7 years	34	5 Mobility Symptoms Sleep Disturbance Everyday activity and pain Participating in everyday life	-	0–100	4	-
Neck Pain and Disability Scale [34]	NPDS	100	44.26 ± 11.07 years	20	0	-	0–100	13	-
Patient Scar Assessment Questionnaire [29]	PSAQ	667	-	28	4 Appearance Scar consciousness Satisfaction with appearance Satisfaction with symptoms	-	112–0	7	-
Patient-Specific Functional Scale 2.0 [35]	PSFS	100	52.6 ± 14.5 years	3	0	-	0–30	5	-
The Neck Bournemouth Questionnaire [32]	NBQ	102	45.4 ± 14.81 years	7	0	-	0–70	10	-
The Northwick Park Neck Pain Questionnaire [19]	NPQ	44	48	9	0	-	0–100	9	-
The Profile Fitness Mapping neck Questionnaire [23]	ProFitMap-neck	N_1_ = 127 N_2_ = 83 N_3_ = 104	39.5 ± 10.5 years 42.9 ± 10.8 years 40.7 ± 9.9 years	44	2 Symptom scale Functional limitation scale	-	100–0	4	-
Total Disability Index [22]	TDI	252	55 years	14	0	-	0–100	2	-
Whiplash Disability Questionnaire [36]	WDQ	66	41.55 ± 12.7 years	13	0	-	0–130	3	-

**Table 2:** Psychometric characteristics of the questionnaires in different versions for neck-pain assessment.

Questionnaire	Test-Retest Reliability (ICC)	Internal Consistency (Cronbach’s α)	Construction Validity	Factor Analysis	Sensitivity	SEM	MDC	MCID
5-item version of the Neck Disability Index	0.91	-	r = 0.67 with NPRS r = 0.54 with TSK-11 r = 0.64 with PCS	-	-	1.15	2.7	-
Cervical Spine Outcomes Questionnaire	0.75–0.86	0.80–0.94	r = 0.36–0.69 with ODI r = −0.20–−0.65 with SF-36	-	-	-	-	-
Copenhagen Neck Functional Disability Scale	0.92	0.90	r = 0.83 with disability and pain scores r = 0.89 patient global assesment	-	-	-	-	-
Dizziness Catastrophizing Scale	0.92	0.95	PANAS negative r = 0.78 PANAS positive r = −0.40	61.6%	-	-	-	-
Dizziness Handicap Inventory	0.97	0.89	-	-	-	6.23	-	18
Fear-Avoidance Beliefs Questionnaire	0.81	0.90	r = 0.33 with NRS; r = 0.53 with NPQ; r = −0.64 SF-36 (physical) r = −0.43 SF-36 (mental)	Factor 1: 40% Factor 2: 11.2% Factor 3: 10.5% Factor 4: 7.4%	-	-	-	-
Functional Rating Index	0.99	0.92	r = 0.71	-	-	2.1	-	-
Hospital Anxiety and Depression Scale	0.54–0.79	-	r = 0.70 for depression r = 0.74 for anxiety	-	-	-	-	-
Neck Disability Index	-	0.80	r = 0.89	-	-	-	-	-
Neck OutcOme Score	0.88–0.95	0.77–0.92	r = 0.23–0.73 with SF-36 r = −0.54–−0.72 with NDI	-	-	5.9–9.54	10–18	-
Neck Pain and Disability Scale	-	0.93	-	Factor 1: 16.529% Factor 2: 18.527% Factor 3: 12.123% Factor 4: 19.424%	-	-	-	-
Patient Scar Assessment Questionnaire	0.73–0.94	0.67–0.87	Appearance: r = 0.25 to 0.71 Consciousness: r = 0.27 to 0.80 Satisfaction with Appearance: r = 0.59 to 0.88 Satisfaction with Symptoms: r = 0.52 to 0.91	-	-	-	-	-
Patient-Specific Functional Scale	0.95	-	r = 0.60 with NDI r = 0.52 with GPE	-	-	0.95−1.25	1.10	2.67
The Neck Bournemouth Questionnaire	0.65	0.87–0.92	r = 0.50 with NDI r = 0.44 with CNFDS	-	-	-	-	-
The Northwick Park Neck Pain Questionnaire	-	0.62	r = 0.84	-	-	-	-	-
The Profile Fitness Mapping neck Questionnaire	0.88–0.96	0.90	r = 0.30–0.59 with SF-36 r ≥ 0.60 with NDI	-	-	0.04	0.12	-
Total Disability Index	0.96	0.922	-	-	-	-	-	-
Whiplash Disability Questionnaire	0.89	-	-	-	-	7.72	21.41	-

MCID: Minimal clinically important difference; MDC: Minimal detectable change; SEM: Standard Error of Measurement.

**Table 3:** Evaluation of the selected PROMs in the present study according to the COSMIN scale regarding the properties that allow assessing methodological quality in the validation process of a patient-reported outcome measure.

PROM	Content Validity	Structural Validity	Internal Consistency	Cross-Cultural Validity	Reliability	Measurement Error	Criterion Validity	Hypothesis Testing	Responsiveness	Total
5-item version of the Neck Disability Index [21]	-	-	-	-	•	•	•	-	-	3
Cervical Spine Outcomes Questionnaire [30]	-	-	•	-	•	-	•	-	-	3
Copenhagen Neck Functional Disability Scale [37]	-	-	•	-	•	-	•	-	-	3
Dizziness Catastrophizing Scale [33]	-	•	•	-	•	-	•	•	•	6
Dizziness Handicap Inventory [24]	-	-	•	-	•	•	-	-	-	3
Fear-Avoidance Beliefs Questionnaire [20]	-	•	•	•	•	-	•	•	•	7
Functional Rating Index [25]	-	-	•	-	•	•	•	•	•	6
Hospital Anxiety and Depression Scale [26]	-	-	-	-	•	-	•	-	-	2
Neck Disability Index [27]	-	-	•	-	•	-	•	•	•	5
Neck OutcOme Score [28]	-	-	•	-	•	•	•	•	•	6
Neck Pain and Disability Scale [34]	-	•	•	-	•	-	-	•	•	5
Patient Scar Assessment Questionnaire [29]	-	-	•	-	•	-	•	•	•	5
Patient-Specific Functional Scale 2.0 [35]	-	-	-	-	•	•	•	•	•	5
The Neck Bournemouth Questionnaire [32]	-	-	•	-	•	-	•	•	•	5
The Northwick Park Neck Pain Questionnaire [19]	-	-	•	-	•	-	•	-	-	3
The Profile Fitness Mapping neck Questionnaire [23]	-	-	•	-	•	•	•	•	•	6
Total Disability Index [22]	-	-	•	-	•	-	-	•	•	4
Whiplash Disability Questionnaire [36]	-	-	-	-	•	•	-	-	-	2

- Negative. • Positive.

## 4. Discussion

The aim of this study was to review the questionnaires related to the assessment of neck pain in their original version to evaluate their structural and psychometric properties.

From all the selected validation studies across different databases, a total of 18 questionnaires were included. These questionnaires exhibit significant heterogeneity in terms of the number of items used, providing various options for selection based on the available time for completion. Regarding psychometric characteristics, internal consistency was the most studied variable, showing good-to-excellent results (Table 2), while MCIC was investigated to a lesser extent, with only two studies assessing it [24,35].

Based on the results, it can be asserted that the objective of the study was achieved.

### 4.1. Structural Variables

With regard to structural characteristics, the great majority of the questionnaires in Table 1 focus on pain and functionality variables [19,21,22,23,25,27,28,29,30,32,34,36,37,38], while others cover different outcome variables, such as dizziness in the case of Dizziness Catastrophizing Scale (DCS) [33] and Dizziness Handicap Inventory (DHI) [24], beliefs about fear and avoidance in Fear-Avoidance Beliefs Questionnaire (FABQ) [20], and anxiety and depression in HADS [26].

Moreover, the number of items in the questionnaires ranges from 5 to 44 questions, with the 5-item version of the Neck Disability Index (NDI-5) [21], NBQ [32] and NPQ [19] presenting the smallest number of items (5, 7 and 9 items, respectively), and ProFitMap-neck [23] and NOOS [28] showing the largest number (44 and 34 items, respectively). In all cases, the items are concentrated in 2-5 subscales, except for the Cervical Spine Outcomes Questionnaire, with 6 subscales [30], and the Neck Disability Index, with 10 subscales [27]. Additionally, all questionnaires have validated versions in other languages, with NDI being the most significant, with 31 versions [27].

Having the same questionnaire adapted and validated in different languages facilitates its use and result comparison among clinical populations with similar characteristics from other countries [22,23,33].

### 4.2. Psychometric Variables

#### 4.2.1. Reliability

Among the 18 identified questionnaires for assessing neck pain, reliability ranges from acceptable to excellent [39], showing values between 0.54 [26] and 0.99 [25], except for the Neck Pain and Disability Scale (NPDS) and NPQ, which do not provide reliability parameters in their original version [19,34]. Furthermore, these questionnaires show good reliability values in other languages, such as the Hausa version of NPQ, with 0.86 [40], the Urdu version of NPQ, with 0.96 [41], the Italian version of NPDS, with 0.942 [42], and the Brazilian version of NPDS, with 0.98 [43]. Thus, these values would indicate that they can be considered good questionnaires to replicate the results obtained from the evaluation process [44].

#### 4.2.2. Internal Consistency

The internal consistency of the analyzed questionnaires ranges between 0.62 [19] and 0.95 [33], with only four studies (HADS, Patient-Specific Functional Scale 2.0 (PSFS), NDI-5, and Whiplash Disability Questionnaire (WDQ)) presenting no data in this respect. Although the translated versions show good Cronbach’s alpha values, with scores of 0.77 for some subscales [45] and 0.91 for the overall scale [46] for HADS, 0.75 for PSFS [47] and 0.89 for WDQ [48], these results demonstrate that these tools favor the stability of the measurement over time, making it easier to compare the result of the intervention over time [49]. They also indicate the good correlation between the different items presented in each questionnaire [50].

#### 4.2.3. Construct Validity

Twelve of the questionnaires used to assess patients with neck pain showed a correlation from low to high, with negative values between −0.20 [30] and −0.72 [28], and positive values between 0.23 [28] and 0.89 [27]. Four questionnaires evaluated their correlation using NDI as a tool for comparison, with positive values ranging from 0.50 to 0.60 [23,28,32] and negative values from −0.54 to −0.72 [35]. This high variability is also evident in translated versions when compared with different assessment tools [51]. Therefore, to thoroughly assess this point, it is necessary to select, as a reference, those questionnaires that have a good value in this area, as the items constituting each questionnaire are representative of the theme to be evaluated [50].

#### 4.2.4. Factor Analysis

Factor analysis was carried out on 3 questionnaires: DCS focused on a single factor with a final result of 61.6% [33], while NPDS and FABQ analyzed multiple factors. For instance, NPDS obtained a value around 20% for each factor [34], and in FABQ, it varied from 7.4% to 40% [20]. In contrast to translated versions, which usually do not present factor analysis, the German version of NDI has a value of 39.8% [52]. This indicates that these questionnaires have a weak or moderate correlation between the variables they measure and the theory that can be established through them [53].

#### 4.2.5. Other Psychometric Properties

No questionnaire presents sensitivity results in its original version, but in versions validated in another language, such as the Thai version of the Functional Rating Index (FRI), which showed 57% [54,55]. Regarding SEM, seven studies present results with 0.04 points in ProFitMap-neck [23], 2.1 in FRI [25], 6.23 in DHI [24], between 5.9 and 9.54 in NOOS [28], between 0.95 and −1.25 in PSFS [35], 1.15 in 5-NDI [21], and 7.72 in WDQ [36], while the versions translated into other languages show differences in the Thai version of FRI, with 0.8 points [54], and in the Chinese version of ProFitMap-neck, ranging between 4.43 and 5.29 [56]. For translated versions into other languages of DHI, this parameter was not evaluated [57,58]. Therefore, the questionnaires show a highly variable range in the sample where the majority of the population is found, considering that higher values may be related to the sample size [59].

The MDC value is reported by ProFitMap-neck, with 0.12 points [23], NOOS, between 10 and 18 points [28], PSFS, with 1.10 points [35], NDI-5, with 2.7 [21], and WDQ, with 21.41 [36]. As for the MCIC value, DHI reported 18 points [24], and PSFS reported 2.67 points [35]. In the different translated versions of these questionnaires, the MDC and MCIC values were not found, but in the Persian version of DHI, 19 points were obtained, and in the Italian version of CNFDS, 8.31 points are reported, whereas the Chinese version of ProFitMap-neck shows a change in percentages ranging from 6.6% to 13.6% [56], and in the Thai version of FRI, 2.5 points are reported [54]. In this regard, it is essential to note that neck pain questionnaires allow assessing patient symptoms with a low score, while determining the significance of a change in the patient usually requires a substantial alteration [60].

#### 4.2.6. Applicability of the Results

In relation to neck pain, several criteria must be considered to select a questionnaire. Without a doubt, the most relevant is the outcome variable that is intended to be measured, although there are other factors that, in a complementary way, should be taken into account, such as the number of items it comprises, the presence of subscales, the average time required for completion, and whether a validated version exists in the language to be self-administered to patients.

For instance, to assess a patient with neck pain and clinical signs related to dizziness, the most commonly used questionnaires are DCS and DHI. DCS can serve as an assessment tool to obtain an initial reference for the patient [33], and DHI can further explore with questions focused on its three subscales: functional, physical, and emotional [24]. If the patient exhibits clinical signs related to anxiety and/or depression, the HADS assessment tool is often employed, as it comprehends the influence of the psychological sphere on the patient’s condition [26]. FABQ may be used for patients with beliefs related to fear and avoidance [20]. Concerning issues related to the patient’s ability to perform tasks, CNFDS assesses neck functionality specifically [37]. Total Disability Index (TDI) combines items from commonly used physiotherapy assessment tools such as NDI and Oswestry Disability Index [22], and FRI, NDI-5, WDQ, CSOQ, and PSFS assess general patient functionality, with WDQ focusing on whiplash injuries and CSOQ additionally assessing pain intensity, distress, physical symptoms, and health care utilization [21,25,30,36,38].

Similarly, ProFitMap-neck is an interesting assessment tool that gathers information on the patient’s disability and how it affects their daily living, using items related to the International Classification of Functioning, Disability and Health [23]. In addition, NBQ, NPDS, NPQ, and NOOS assess the impact of pain on the patient’s life and are considered easy for patients to use and understand [19,28,32,34]. On the other hand, PSAQ is employed to assess the patient’s perception of scars and symptoms following neck surgery [29], and, despite being the most widely used questionnaire in clinical and research settings, NDI provides a comprehensive, simple, and few-item assessment of neck pain stratified into different categories [27].

Among the questionnaires specific to patients with neck pain, several of them ask questions related to the patient’s pathology and its impact [19,22,32,34,37]. Other questionnaires have subscales to assess the degree of symptom involvement, functionality, daily activities, and even the patient’s thoughts [21,23,25,27,29,30].

Out of all the questionnaires, only two validation studies focused on evaluating the completion time, with approximately 78 s used to fill out 10 items in FRI, suggesting an average of 7–8 s per item [25], and about 5 min to complete 11 items in FABQ, at a rate of almost 30 s per item [20].

Similarly, considering the number of questionnaires validated in different languages, which, as previously noted, would allow comparing between the same type of patients across different clinical environments [27], NDI is the questionnaire with the largest number of translated and validated versions in other languages, which is considered a significant advantage for an assessment tool in clinical and research settings.

To summarize, having all the questionnaires that are responsible for assessing the cervical region allows clinicians and researchers to select the assessment and monitoring tool that best suits their clinical needs or objectives, ranging from the most general and well-known for its clinical and research use, such as NDI, to the most specific, such as DHI and FABQ, and even to complement or cross-referencing data between them to provide a more general view of what is happening to the patient. The selection criteria can be very varied, including the outcome variable and time to complete, among others.

#### 4.2.7. Limits on the Design of Validation Studies

Despite the fact that all the included studies were designed for their intended purpose and exhibit good values in their psychometric properties, it is important to note that most of them present poor methodological quality. However, in the cross-cultural validation of some versions of the selected PROMs, it was observed that some of these domains have been calculated. In this regard, it is important to note that some very important psychometric variables have not been calculated by any of the selected PROMs, such as sensitivity. Additionally, others have been calculated by a very limited number of PROMs, e.g., SEM [7,18], MDC [5,18], and MCID [2,18]. Therefore, it would be necessary for the original versions to undergo a study with a design that allows calculating these important psychometric characteristics. These problems could be due to the scarcity of assessment of all psychometric properties [61], and, in other words, to the low methodological quality of the studies themselves [62], which is also reflected in their translated versions [63].

All of this highlights the importance of using the COSMIN checklist to drive the development of validation studies, rather than simply designing new assessment tools [9]. Additionally, given that most questionnaires included in this study were designed over 20 years ago, changes in sample characteristics and population development might lead to differences in comprehension. This suggests the need to design and conduct new studies aimed at calculating the missing psychometric variables. Doing so would provide tools that allow not only for a one-time patient evaluation but also for ongoing monitoring to identify changes in the patient as a result of clinical intervention.

### 4.3. Strengths and Limitations

To the best of our knowledge, no systematic reviews have thoroughly analyzed the psychometric and structural properties of all questionnaires published to date for the assessment and monitoring of neck pain patients, and there are two systematic reviews that analyze them to a lesser extent [64,65]. Nevertheless, only three questionnaires assessed pain and functionality outcomes in this systematic review [61,63,66], not only from a biological perspective but also introducing psychosocial aspects, allowing both clinicians and researchers to have a broad understanding of what is happening to the patient. Another noteworthy point is the good reliability and internal consistency of each of the questionnaires, providing robustness to the results obtained from the patients.

However, certain limitations should be considered, such as the fact that this study did not conduct a more globally comprehensive search (Pubmed, Scopus, Cochrane Library, Trip Medical Database); thus, there may exist validated studies in other databases that were not identified and were excluded from the results of this study. Furthermore, the languages selected for the inclusion of studies were Spanish, English, and Italian. While these three languages cover a very important population spectrum, especially English, which traditionally constitutes the backbone of scientific language worldwide, there are other languages with a significant population impact. Examples include Chinese, Japanese, German, and French, among others, which were not included in this study. These languages may offer complementary tools that could enrich the results of the current study, or even address some of the limitations in the design of validation studies identified in this research.

## 5. Conclusions

According to the data presented in this review, neck pain questionnaires exhibit good structural and psychometric characteristics. Despite presenting heterogeneity in their structural characteristics, the standardization of the ranges for the results tends to show a certain homogeneity. Moreover, the psychometric characteristics of all questionnaires generally demonstrate moderate-to-good overall values. This implies that healthcare professionals have several suitable assessment tools to objectively assess patients with neck pain. When selecting a PROM, it is important to have a very clear idea of which criteria will be prioritized for the selection. The primary criterion (for example, the main outcome variable) should be identified, although complementary aspects must also be considered if there are different options, such as the number of items, completion time, secondary variables, and so on.

Future studies should calculate the psychometric variables that have not been analyzed in many of the PROMs selected in this study, specifically, factor analysis, sensitivity, Standard Error of Measurement (SEM), MDC, and MCIC, even if there are validated versions in other languages where these variables are thoroughly studied and good values are obtained. Lastly, it would be beneficial to standardize and homogenize the assessment of psychometric data in order to reduce the scarcity of questionnaire information for both clinicians and researchers.

## Data Availability

Data are available upon request.

## Supplementary Materials

The following supporting information can be downloaded at: https://www.mdpi.com/article/10.3390/medicina61071254/s1, Table S1: Search Strategy; Table S2: Risk of bias.

## Abbreviations

The following abbreviations are used in this manuscript:

CNFDS	Copenhagen Neck Functional Disability Scale
COSMIN	COnsensus-based Standards for selecting health Measurement Instruments
CSOQ	Cervical Spine Outcomes Questionnaire
DALYS	Disability-adjusted life years
DCS	Dizziness Catastrophizing Scale
DHI	Dizziness Handicap Inventory
FABQ	Fear-Avoidance Beliefs Questionnaire
FRI	Functional Rating Index
HADS	Hospital Anxiety and Depression Scale
ICF	International Classification of Functioning, Disability and Health
NBQ	Neck Bournemouth Questionnaire
NDI	Neck Disability Index
NOOS	Neck OutcOme Score
NDI-5	5-item version of the Neck Disability Index
NPDS	Neck Pain and Disability Scale
NPQ	Northwick Park Neck Pain Questionnaire
MCID	Minimal Clinically Important Difference
MDC	Minimal Detectable Change
PRISMA	Preferred Reporting Items for Systematic Reviews and Meta-analyses
ProFitMap-neck	Profile Fitness Mapping neck Questionnaire
PROM	Patient-reported outcome measure
PSAQ	Patient Scar Assessment Questionnaire
PSFS	Patient-Specific Functional Scale 2.0
SF-36	Short Form-36 Health Survey
TDI	Total Disability Index
SEM	Standard Error of Measurement
WDQ	Whiplash Disability Questionnaire
