# Supplementary material for: Structural and Psychometric Properties of Neck Pain Questionnaires Through Patient-Reported Outcome Measures: A Systematic Review
Source: Medicina (Kaunas). 2025 Jul 10;61(7):1254. doi: 10.3390/medicina61071254 (PMC12300700; doi:10.3390/medicina61071254)
Supplement: Supplementary file 1 [file medicina-61-01254-s001.zip › Appendix I-Search Strategy.pdf]

DATABASE	SEARCH STRATEGY
Pubmed	(neck pain OR neckache or neck injur* OR cervicalgia OR cervical pain OR whiplash injuries) AND (questionnaire OR survey OR index OR scale OR tool OR inventory OR instrument OR score OR checklist OR PROM OR Patient Reported Outcome Measure) AND (validity OR validation OR reliability OR psychometric properties OR clinimetric*)
Cochrane Library	(neck pain OR neckache or neck injur* OR cervicalgia OR cervical pain OR whiplash injuries) AND (questionnaire OR survey OR index OR scale OR tool OR inventory OR instrument OR score OR checklist OR PROM OR Patient Reported Outcome Measure) AND (validity OR validation OR reliability OR psychometric properties OR clinimetric*)
EMBASE	(neck pain OR neckache or neck injur* OR cervicalgia OR cervical pain OR whiplash injuries) AND (questionnaire OR survey OR index OR scale OR tool OR inventory OR instrument OR score OR checklist OR PROM OR Patient Reported Outcome Measure) AND (validity OR validation OR reliability OR psychometric properties OR clinimetric*)
CINHAL	(neck pain OR neckache or neck injur* OR cervicalgia OR cervical pain OR whiplash injuries) AND (questionnaire OR survey OR index OR scale OR tool OR inventory OR instrument OR score OR checklist OR PROM OR Patient Reported Outcome Measure) AND (validity OR validation OR reliability OR psychometric properties OR clinimetric*)
Trip Medical Database	(neck pain OR neckache or neck injur* OR cervicalgia OR cervical pain OR whiplash injuries) AND (questionnaire OR survey OR index OR scale OR tool OR inventory OR instrument OR score OR checklist OR PROM OR Patient Reported Outcome Measure) AND (validity OR validation OR reliability OR psychometric properties OR clinimetric*) Filters: Title, Abstract or Author keywords
Scopus	(neck pain OR neckache or neck injur* OR cervicalgia OR cervical pain OR whiplash injuries) AND (questionnaire OR survey OR index OR scale OR tool OR inventory OR instrument OR score OR checklist OR PROM OR Patient Reported Outcome Measure) AND (validity OR validation OR reliability OR psychometric properties OR clinimetric*) Filters: Article title, Abstract & Keywords
