# Supplementary material for: Structural and Psychometric Properties of Neck Pain Questionnaires Through Patient-Reported Outcome Measures: A Systematic Review
Source: Medicina (Kaunas). 2025 Jul 10;61(7):1254. doi: 10.3390/medicina61071254 (PMC12300700; doi:10.3390/medicina61071254)
Supplement: Supplementary file 1 [file medicina-61-01254-s001.zip › Appendix II-Risk of bias.pdf]

PROM	Content validity	Structural validity	Internal consistency	Cross-cultural validity	Reliability	Measurement error	Criterion validity	Hypothesis testing	Responsiveness
Copenhagen Neck Functional Disability Scale (37)	-	-	Very good	-	Adequate	-	-	Inadequate	-
Dizziness Catastrophizing Scale (33)	-	Very good	Very good	-	Doubtful	-	Very good	Adequate	Adequate
Dizziness Handicap Inventory (24)	-	-	Very good	-	Doubtful	Doubtful	Very good	Inadequate	Inadequate
Fear-Avoidance Beliefs Questionnaire (20)	Inadequate	Very good	Very good	Adequate	Adequate	-	Very good	Very good	Very good
Functional Rating Index (25)	-	Inadequate	Very good	-	Adequeate	Very good	Very good	Doubtful	Doubtful
Hospital Anxiety and Depression Scale (26)	-	Inadequate	Doubtful	-	Doubtful	-	Very good	Inadequate	Inadequate
Neck Disability Index (27)	-	Inadequate	Very good	-	Doubtful	-	Very good	Adequate	Adequate
Neck OutcOme Score (28)	-	Inadequate	Very good	-	Very good	Very good	Very good	Very good	Very good
Neck Pain and Disability Scale (34)	-	Inadequate	Very good	-	Doubtful	-	Very good	Adequate	Adequate
Patient Scar Assessment Questionnaire (29)	-	Inadequate	Very good	-	Adequate	-	Very good	Very good	Very good
Patient-Specific Functional Scale 2.0 (35)	-	Inadequate	Doubtful	-	Adequate	Very good	Very good	Very good	Very good

The Neck Bournemouth Questionnaire (32)	-	Inadequate	Very good	-	Adequate	-	Very good	Adequate	Adequate
The Northwick Park Neck Pain Questionnaire (19)	-	Inadequate	Very good	-	Doubtful	-	Very good	Inadequate	Inadequate
The Profile Fitness Mapping neck Questionnaire (23)	-	Inadequate	Very good	-	Adequate	Adequate	Very good	Doubtful	Doubtful
Total Disability Index (22)	-	Inadequate	Very good	-	Adequate	-	Very good	Doubtful	Doubtful
Cervical Spine Outcomes Questionnaire (30)	-	-	Very good	-	Adequate	-	Inadequate	Inadequate	Inadequate
5-item version of the Neck Disability Index (21)	-	Inadequate	Inadequate	-	Adequate	Adequate	Inadequate	Inadequate	Inadequate
Whiplash Disability Questionnaire (36)	-	Inadequate	-	-	Very good	Very good	Inadequate	Inadequate	Inadequate
